# Malaria Elimination in Suriname, Timor‐Leste and Georgia: Strategic Lessons for Sub‐Saharan Africa

**DOI:** 10.1155/jotm/2927281

**Published:** 2026-09-02

**Authors:** Christian Tague, Aymar Akilimali, Dujardin Makeda, Hermann Yokolo, Joshua Ekouo, Germain Cebweru Murhola, Johan Domga, Josias Silatchom Kamgang, Criss Koba Mjumbe

**Affiliations:** ^1^ Department of Research, Medical Research Circle (MedReC), Goma, Democratic Republic of the Congo; ^2^ Centre de Recherche et de d’Appui Aux Systèmes de Santé (CRASS), Goma, Democratic Republic of the Congo; ^3^ Faculty of Medicine, Université Libre des Pays des Grands Lacs, Goma, Democratic Republic of the Congo; ^4^ Department of Public Health, Faculty of Medicine, University of Lubumbashi, Lubumbashi, Democratic Republic of the Congo, unilu.ac.cd

**Keywords:** elimination, epidemiological surveillance, Georgia, global health, malaria, political commitment, ub-Saharan Africa, Suriname, Timor-Leste

## Abstract

**Background:**

Malaria remains a major cause of morbidity and mortality worldwide, with over 263 million cases and 597,000 deaths estimated in 2023, of which 94% occurred in Sub‐Saharan Africa. Despite this heavy burden, several low‐ and middle‐income countries have recently succeeded in eliminating local malaria transmission.

**Objective:**

This article describes and synthesises malaria elimination experiences in Suriname, Timor‐Leste and Georgia, all three certified malaria‐free by the World Health Organization in 2025, to identify strategic lessons and contextual factors that may inform elimination efforts in malaria‐endemic countries, including those in Sub‐Saharan Africa.

**Methodology:**

A structured narrative review was conducted using official reports from WHO, the Global Fund and PAHO; national public health documents; and peer‐reviewed articles indexed in PubMed, Scopus and Google Scholar between 2012 and 2025. Search terms included ‘malaria elimination,’ ‘malaria‐free certification,’ ‘Suriname malaria,’ ‘Timor‐Leste malaria’ and ‘Georgia malaria.’ Inclusion criteria required relevance to elimination strategies, publication in English or French and availability in full text. Grey literature from WHO, PAHO and the Global Fund was systematically included.

**Results:**

The three countries share several common determinants of success: strong and sustained political commitment, robust epidemiological surveillance systems, active community mobilisation and consistent financial support. Strategies were adapted to context: involvement of indigenous communities and cross‐border control in the Amazon basin (Suriname), universal coverage and extensive partner support in a postconflict setting (Timor‐Leste) and institutional resilience and postelimination vigilance in a context of political transition (Georgia).

**Conclusion:**

These experiences demonstrate that malaria elimination is achievable even in resource‐limited or fragile contexts, provided interventions are tailored to the local setting and supported by coherent governance. Key lessons for malaria‐endemic countries include strengthening community‐based surveillance, cross‐border cooperation, community education and securing sustainable financing. The risk of reintroduction remains real, and postelimination vigilance is essential.

**Highlights**
• In 2023, malaria caused an estimated 263 million cases and 597,000 deaths, of which 94% occurred in sub‐Saharan Africa, confirming its status as a major global health threat.
• Three countries certified malaria‐free by WHO in 2025 Suriname, Timor‐Leste and Georgia demonstrate that elimination is achievable across diverse geographic and socioeconomic contexts.
• Determinants of success include sustained political commitment, robust epidemiological surveillance, community mobilisation and stable funding.
• Each experience illustrates context‐specific adaptations: cross‐border control and indigenous community engagement in the Amazon; universal coverage in a post‐conflict setting; institutional resilience in a post‐Soviet country.
• Key lessons for malaria‐endemic settings include strengthening community‐based surveillance, cross‐border cooperation, community education and securing sustainable financing.

## 1. Introduction

Malaria, a parasitic disease transmitted by female *Anopheles* mosquitoes, remains a major public health challenge. Despite remarkable progress over the past two decades, it continues to cause millions of cases and hundreds of thousands of deaths each year, mainly in Sub‐Saharan Africa and South‐East Asia [1, 2]. In response, the World Health Organization (WHO) established an ambitious Global Technical Strategy for 2016–2030, aiming to reduce incidence and mortality by at least 90% and to eliminate the disease in at least 35 countries by 2030 [3].

In this context, 2025 represents a significant milestone, with WHO certifying three countries as malaria‐free: Suriname in South America, Timor‐Leste in South‐East Asia and Georgia in Eastern Europe [4–6]. These three countries were selected for analysis because they represent the most recently certified malaria‐free nations, each from a distinct WHO region, offering diverse epidemiological, political and socioeconomic contexts. Their experiences illustrate that malaria elimination is not determined solely by economic development, but by the combination of context‐adapted strategies, sustained political commitment and effective community mobilisation.

While a growing body of literature documents malaria elimination in various settings, no comparative analysis has been published for these three countries certified concurrently in 2025. Understanding their shared determinants of success and contextual adaptations could provide actionable insights for countries where malaria remains endemic, particularly in Sub‐Saharan Africa where more than 94% of the global burden is concentrated.

This article aims to describe and synthesise recent malaria elimination experiences in Suriname, Timor‐Leste and Georgia, in order to identify key strategies, contextual factors, persistent challenges and lessons that may inform efforts to achieve the WHO 2030 malaria elimination targets.

## 2. Methodology

### 2.1. Study Design

This study used a structured narrative review design to synthesise published and grey literature on malaria elimination in three recently WHO‐certified countries: Suriname (June 2025), Timor‐Leste (July 2025) and Georgia (January 2025). Although the study is primarily descriptive, a comparative analytical framework was applied to identify commonalities, contextual differences and transferable lessons across the three cases.

### 2.2. Rationale for Country Selection

The three countries were selected because: (i) they represent the three most recently certified malaria‐free countries at the time of writing, all certified in 2025; (ii) they span three distinct WHO regions (Americas, South‐East Asia and Europe), providing geographic diversity; and (iii) they represent different socioeconomic and epidemiological contexts, enabling a comparative perspective. Other recently certified countries such as Cabo Verde (2024) were not included because their certification predated the 2025 milestone that motivated this review.

### 2.3. Search Strategy and Sources

A structured narrative literature search was conducted in PubMed, Scopus and Google Scholar in August 2025. The search covered publications from January 2012 to August 2025 and was supplemented by targeted searches of WHO, PAHO, Global Fund and relevant national public health institution websites.

The search combined terms related to malaria elimination, malaria‐free certification, surveillance, vector control, cross‐border transmission and Sub‐Saharan Africa. The main search strategy included (‘malaria elimination’ OR ‘malaria‐free certification’) AND (‘Suriname’ OR ‘Timor‐Leste’ OR ‘Georgia’).

A total of 366 records were retrieved from PubMed (*n* = 88), Scopus (*n* = 114) and Google Scholar (*n* = 164). After removal of 72 duplicate records, 294 titles and abstracts were screened. 221 records were excluded because they were unrelated to malaria elimination or did not meet the eligibility criteria. 73 full‐text documents were assessed for eligibility, of which 17 were excluded (e.g., insufficient data, duplicate reports, conference abstracts or not focused on elimination/certification). Finally, 56 references were included in the narrative synthesis, comprising peer‐reviewed publications, institutional reports and official national documents.

### 2.4. Inclusion and Exclusion Criteria

Documents were included if they (i) focused on malaria control or elimination strategies in Suriname, Timor‐Leste, Georgia or Sub‐Saharan Africa; (ii) were published in English or French; (iii) were available in full text; and (iv) represented peer‐reviewed articles, official institutional reports or national public health documents. Documents were excluded if they were opinion pieces lacking epidemiological data, studies not relevant to the elimination context or publications outside the 2012–2025 time frame unless providing essential historical context.

### 2.5. Data Extraction and Synthesis

Data extraction focused on epidemiological indicators (case incidence trends, burden at peak and at elimination), key interventions deployed, community and institutional actors involved, cross‐border and surveillance mechanisms, funding sources and contextual factors. Findings were synthesised thematically and presented through a comparative framework across the three countries. A summary table was constructed to facilitate cross‐country comparison.

Evidence was prioritised according to source credibility. Peer‐reviewed publications were considered the primary source of scientific evidence. Official reports from WHO, PAHO, the Global Fund and national malaria programmes were used to complement recent epidemiological data and policy information.

## 3. Results

### 3.1. Global Burden of Malaria, With a Focus on Sub‐Saharan Africa

In 2023, an estimated 263 million cases of malaria were recorded worldwide, resulting in approximately 597,000 deaths [7]. Africa remains the most affected region, accounting for 94% of cases (approximately 246 million) and 95% of deaths (approximately 569,000) [2, 8]. Children under five years of age represent the most vulnerable population, accounting for approximately 76% of malaria‐related deaths in the African region [2, 9]. The malaria burden is particularly concentrated in five countries: Nigeria, the Democratic Republic of Congo (DRC), Uganda, Ethiopia and Mozambique, which collectively account for more than half of global cases. Nigeria alone accounts for approximately 31% of global malaria deaths, followed by the DRC (approximately 11%), Niger (approximately 6%) and Tanzania (approximately 4%) [2, 9, 10].

Recent years have seen worrying setbacks in some countries. Between early 2024 and mid‐2025, Zimbabwe experienced a marked resurgence of malaria, with deaths tripling from 45 to 143 and more than 115 outbreaks involving nearly 120,000 cases, partly driven by delays in external funding [11–13]. In the DRC, the Equateur Province was affected by a severe epidemic in early 2025, with approximately 1100 suspected cases and 60 deaths, reflecting a case fatality rate of approximately 6.3% [14–16].

At the same time, important progress has been made. As of March 2025, malaria vaccines were being deployed in 18 African countries. Newer, more effective insecticide‐treated nets now account for more than 80% of the 195 million nets distributed across Africa. Seasonal malaria chemoprevention expanded from 170,000 children protected per cycle in 2012 to 53 million in 2023 [17–19].

### 3.2. Suriname: An Amazonian Model of Context‐Adapted Elimination

Suriname became the first country in the Amazon region to be certified malaria‐free by WHO, in June 2025 [4, 20, 21]. Malaria elimination in this country occurred in a particularly complex context: while the coastal area had benefited from DDT spraying since the 1960s, the interior, more than 90% covered by Amazonian rainforest and inhabited by indigenous communities (Amerindians and Maroons), remained an area of high transmission, sustained by population mobility associated with artisanal gold mining and cross‐border movements [22–24].

The national malaria incidence declined progressively, from high transmission levels in the interior during the 1990s and early 2000s to near‐zero indigenous cases by the early 2010s. Three strategic pillars underpinned this success. First, vector control evolved from indoor residual spraying (IRS), which proved poorly adapted to traditional open‐construction dwellings, toward the mass distribution of insecticide‐treated mosquito nets targeting the most exposed communities [24, 25]. Second, the decentralisation of malaria control to Medische Zending, a primary care provider with deep roots in remote communities, enabled the training of village health workers since the 1970s. From 2006 onwards, a network of Malaria Service Deliverers provided free diagnostic, treatment and prevention services in artisanal mining camps, irrespective of the legal status of mobile populations [22, 24]. Third, cross‐border surveillance was strengthened through systematic screening at border crossings and case investigation supported by rapid diagnostic tests (RDTs) and improved microscopy, within the broader framework of the Amazon Malaria Initiative (AMI), supported by the Global Fund, WHO/PAHO and the United States government [22, 24].

### 3.3. Timor‐Leste: Universal Coverage and Partner Commitment in a Post‐Conflict Setting

Timor‐Leste, a young nation still rebuilding from prolonged armed conflict, was certified malaria‐free by WHO in July 2025, having recorded more than 223,000 cases in 2006 at peak transmission. Indigenous malaria transmission was interrupted by 2021, marking a major public health milestone for this small island state [5, 26, 27].

The country’s elimination pathway rested on four main components. In 2003, the Ministry of Health launched a National Malaria Control Programme, enabling the rapid deployment of RDTs, artemisinin‐based combination therapies (ACTs) and the free distribution of insecticide‐treated nets to vulnerable populations [5, 28, 29]. From 2009 onwards, Global Fund support enabled intensified interventions, including mass net distribution campaigns, IRS and the expansion of diagnostic capacity throughout the health system, including peripheral health posts [30, 31]. Despite limited human resources, investment in the health system created a structured network of community health centres and health posts, supplemented by mobile clinics to reach remote populations. Free access to diagnostics and treatment ensured that most patients could obtain care within approximately 1 hour’s travel from their homes [5, 32–34]. Finally, strong political will combined with intersectoral coordination, integrated epidemiological surveillance and sustained community engagement formed the backbone of the national strategy.

### 3.4. Georgia: Institutional Resilience and Postelimination Vigilance in a Post‐Soviet Context

Georgia experienced a resurgence of malaria in the 1990s following the collapse of health infrastructure after the dissolution of the Soviet Union, despite the disease having been eliminated nationally by the 1970s. Cases peaked at 437–474 per year in 2001‐2002. Indigenous transmission was interrupted by 2010, and WHO certified Georgia as malaria‐free in January 2025 [35–37].

The Georgian elimination strategy centred on three axes. First, strengthening epidemiological surveillance was central: the Centre for Disease Control and Public Health (CDC), in collaboration with WHO and the Global Fund, recalibrated case detection and monitoring systems, enabling rapid and targeted response to active foci [35, 37, 38]. Second, regional collaboration was reinforced through Georgia’s participation in the Tashkent Declaration (2005) and the Ashgabat Statement (2017), both aimed at sustaining elimination and preventing reintroduction, particularly via cross‐border migratory flows [6, 35, 39]. Third, stable funding and institutional sustainability were key: consistent resources maintained a resilient health system capable of ensuring quality diagnosis, treatment and postelimination surveillance [6, 35, 37]. Postelimination surveillance metrics showed zero indigenous cases from 2010 to certification in 2025, with annual imported case investigations documenting no secondary transmission (Table 1).

**TABLE 1:** Comparative overview of malaria elimination strategies in Suriname, Timor‐Leste and Georgia.

Domain	Suriname	Timor‐Leste	Georgia
WHO Certification	June 2025	July 2025	January 2025
Malaria burden (peak)	High interior transmission (pre‐2000s)	> 223,000 cases (2006)	437–474 cases (2001–02)
Indigenous transmission interrupted	∼2010s	2021	2010
Key vector control	IRS + ITNs (adapted to open dwellings)	ITNs + IRS mass campaigns	Targeted IRS + case detection
Surveillance model	Border screening + RDTs in mining camps	Integrated facility + mobile clinics	CDC‐led focal surveillance
Community engagement	Medische Zending + Malaria Service Deliverers	Community health centres + mobile teams	National CDC + regional partners
Cross‐border collaboration	Amazon Malaria Initiative (PAHO/Global Fund/USAID)	Indonesia border coordination	Tashkent Declaration; Ashgabat Statement
Key financing	Global Fund + USAID	Global Fund	Global Fund + national budget
Main contextual challenge	Amazonian geography + gold mining mobility	Postconflict reconstruction, limited HRH	Post‐Soviet political transition

Abbreviations: CDC, Centre for Disease Control; HRH, Human Resources for Health; IRS, indoor residual spraying; ITNs, insecticide‐treated nets; RDTs, rapid diagnostic tests.

## 4. Discussion

### 4.1. Cross‐Cutting Determinants of Success

Despite profoundly different geographic, political and socioeconomic contexts, the three countries share several cross‐cutting determinants of malaria elimination success: strong and sustained political commitment, robust epidemiological surveillance systems, active community mobilisation and consistent international financial support. These findings align with existing literature on malaria elimination in other settings, including Sri Lanka, Paraguay and El Salvador, where similar combinations of political will and adaptive strategy implementation have been identified as essential [40, 41].

### 4.2. Contextual Adaptations and Their Relevance

A key finding of this review is that no single elimination model is universally transferable. Suriname’s success depended on reaching highly mobile, geographically isolated populations through decentralised, community‐embedded service delivery, an approach less applicable to high‐density urban malaria contexts common in Sub‐Saharan Africa. Timor‐Leste’s pathway relied on substantial international financial flows through the Global Fund at a scale that may not be sustained indefinitely. Georgia’s model, while highly instructive regarding postelimination vigilance, was implemented in a context of low transmission and a relatively functional (though damaged) health infrastructure.

For Sub‐Saharan Africa, where malaria burden is vastly higher and transmission more intense, the direct application of these models requires careful contextual adaptation. Nevertheless, several principles remain relevant regardless of context: the importance of community health worker networks for surveillance and treatment access, the necessity of sustained cross‐border coordination to prevent case importation, the value of integrated data systems for real‐time decision‐making and the centrality of stable and predictable financing.

For Sub‐Saharan Africa, the main lesson is therefore to adapt strategic principles rather than replicate individual country models. Community‐based surveillance may be particularly relevant for remote and mobile populations, while cross‐border coordination is essential where population movement sustains the risk of malaria importation. Long‐term financing and strong health systems remain necessary to maintain interventions as transmission declines. The applicability of these lessons will nevertheless depend on local transmission intensity, population mobility, ecological conditions and health‐system capacity.

### 4.3. Comparative Lessons and Transferability

Across the three countries, several common success factors consistently emerged despite substantial contextual differences. These included sustained political commitment, continuous financial investment, strong surveillance systems capable of rapidly detecting and responding to imported cases and active community participation. All three countries also maintained close collaboration with WHO and international partners throughout the elimination process.

However, several operational challenges differed considerably. Suriname faced persistent cross‐border transmission linked to mobile mining populations and difficult access to remote rainforest communities. Timor‐Leste had to rebuild essential health services following prolonged conflict while maintaining nationwide intervention coverage. Georgia primarily addressed the challenge of preventing malaria re‐establishment after elimination through long‐term surveillance and regional cooperation.

Importantly, not all successful interventions can be directly transferred to high‐transmission African settings. The epidemiological burden, population size, vector ecology and health‐system constraints differ substantially from those observed in the three certified countries. Rather than replicating individual interventions, endemic African countries should adapt the underlying strategic principles, including surveillance strengthening, community engagement, cross‐border collaboration and sustainable financing, to their own epidemiological context.

### 4.4. Prospects for Sub‐Saharan Africa

For Sub‐Saharan Africa, where the challenges are compounded by insecticide and antimalarial drug resistance, climate change, fragile health systems and persistent funding gaps [42–45], four priority areas emerge from this comparative analysis as follows:

1. Strengthening local, community‐based surveillance: Rwanda’s Community Health Information System (CHIS), which enables weekly case reporting with real‐time stock alerts, offers a replicable model [46].
2. Developing cross‐border cooperation: bi‐ and tri‐national initiatives such as the Trans‐Kunene Malaria Initiative, the Lubombo Spatial Development Initiative (LSDI) and the MOSASWA programme illustrate how regional collaboration can reinforce national responses [45, 47].
3. Investing in community education and ownership: involvement of community leaders, schools, faith‐based organisations and local structures improves uptake of preventive measures and sustains behavioural change [45, 48].
4. Securing sustainable and diversified financing: beyond donor dependence, domestic resource mobilisation, private sector engagement and integration of malaria into broader development frameworks are essential to long‐term programmatic sustainability [19, 49, 50].

## 5. Conclusion

The malaria elimination experiences of Suriname, Timor‐Leste and Georgia demonstrate that eliminating malaria is not a goal restricted to high‐income countries, but an achievable objective even in settings characterised by socioeconomic or political challenges. Shared determinants of success across these three contexts include sustained political commitment, robust surveillance systems, community mobilisation and reliable financing. Their experiences offer instructive, if not directly transferable, lessons for malaria‐endemic countries worldwide, including those in Sub‐Saharan Africa.

However, the lessons from these three countries cannot be straightforwardly applied to the African context, where transmission intensity, population size, ecological diversity and systemic health challenges differ substantially. Context‐specific adaptation of strategies is essential. For countries working toward malaria elimination, these examples underscore the importance of tailoring interventions, maintaining postelimination vigilance given that the risk of reintroduction remains real and ensuring that health systems remain resilient and capable of rapid response.

## 6. Limitations

Several limitations should be acknowledged. First, this review uses a structured narrative design, which, while allowing synthesis across diverse sources, does not involve a formal quality appraisal of individual studies, as would be required in a systematic review. The findings are therefore descriptive and synthesising rather than inferential. Second, a selection bias inherent to the study design may be noted: the review focuses exclusively on countries that successfully achieved WHO‐certified elimination; unsuccessful or incomplete elimination attempts are not examined, which limits the identification of risk factors for failure. Third, the three countries examined present epidemiological, demographic and health‐system profiles that differ substantially from most Sub‐Saharan African settings, limiting the direct transferability of conclusions. Fourth, the reference list combines peer‐reviewed publications, institutional reports and a small number of media sources; the quality and level of evidence associated with each source type is heterogeneous. Fifth, researchers or public health experts from Suriname, Timor‐Leste and Georgia were not involved in the design, data extraction, or validation of findings, which may limit the contextual accuracy of interpretations. Finally, the absence of a documented PRISMA flow diagram means that full reproducibility of the search and selection process cannot be guaranteed.

**Nomenclature**
ACTs: Artemisinin‐based combination therapies
ALMA: African Leaders Malaria Alliance
AMI: Amazon Malaria Initiative
CDC: Centre for Disease Control
CHIS: Community Health Information System
DDT: Dichlorodiphenyltrichloroethane
DRC: Democratic Republic of Congo
GFATM: Global Fund to Fight AIDS, Tuberculosis and Malaria
IRS: Indoor residual spraying
ITNs: Insecticide‐treated nets
LSDI: Lubombo Spatial Development Initiative
MOSASWA: Mozambique–South Africa–Swaziland cross‐border malaria initiative
PAHO: Pan American Health Organization
RDTs: Rapid diagnostic tests
USAID: United States Agency for International Development
WHO: World Health Organization

## Funding

No funding was used in this study.

## Ethics Statement

The authors have nothing to report.

## Consent

The authors have nothing to report.

## Conflicts of Interest

The authors declare no conflicts of interest.

## Data Availability

Data sharing is not applicable to this article as no datasets were generated or analysed during the current study.
